# Echocardiographic Documentation of Dilated Cardiomyopathy Development in Dogs Naturally Infected with *Trypanosoma cruzi*

**DOI:** 10.3390/ani14131884

**Published:** 2024-06-26

**Authors:** Eduardo E. Avalos-Borges, Carlos M. Acevedo-Arcique, Jose C. Segura-Correa, Matilde Jiménez-Coello, Nisha J. Garg, Antonio Ortega-Pacheco

**Affiliations:** 1 Departamento de Salud Animal y Medicina Preventiva, Facultad de Medicina Veterinaria y Zootecnia, Universidad Autónoma de Yucatán, Km 15.5 Carretera Mérida-Xmatkuil, Apdo. Postal 4-116 Itzimná, Mérida 97000, Yucatán, Mexico; animal.health@hotmail.com (E.E.A.-B.); jose.segura@correo.uady.mx (J.C.S.-C.); 2 Hospital Veterinario para Perros y Gatos, Universidad Autónoma de Yucatán, Av. Itzaes No. 490 x 29, C. 18 No. 271, San José Vergel, Mérida 97000, Yucatán, Mexico; carlos.acevedo@correo.uady.mx; 3 Laboratorio de Microbiologia, Centro de Investigaciones Regionales “Dr. Hideyo Noguchi”, Universidad Autónoma de Yucatán, Av. Itzaes No. 490 x 29, Mérida 97000, Yucatán, Mexico; mjcoello@correo.uady.mx; 4 Department of Microbiology & Immunology, University of Texas Medical Branch, Galveston, TX 77555-1070, USA; nigarg@utmb.edu

**Keywords:** echocardiographic alterations, left ventricle, dog, *Trypanosoma cruzi*, flow patterns, dilated cardiomyopathy, Chagas disease

## Abstract

**Simple Summary:**

*Trypanosoma cruzi* is a protozoan parasite with high tropism to the cardiac tissue, causing severe cardiopathies. This study determined the changes in the cardiac structure and myocardial parameters using real-time ultrasonography in dogs naturally infected with *T. cruzi* while living in an endemic region. A major indicator of cardiac involvement in infected dogs was changes in the left ventricular internal diameter (LVID) during systole and diastole. Changes in the intraventricular septum and LV posterior wall (LVPW) thickness at systole and diastole, as well as findings of fractional shortening and an E/A ratio above or below the normal range, may be used to predict dilated cardiomyopathy (DCM) progression.

**Abstract:**

We aimed to characterize the echocardiographic alterations in dogs from an endemic region that were naturally infected with *T. cruzi*. Dogs (n = 130) seropositive for antibodies against *T. cruzi* and/or with acute parasitemia were enrolled in the study. Indicators of changes in the structure and systolic and diastolic functions of the left ventricle (LV) and blood flow patterns were evaluated by echocardiography. The frequency and extent of alterations in these indicators were associated with the severity of the disease. Briefly, 15 (11.54%) dogs were diagnosed with dilated cardiomyopathy (DCM), and 115 (88.46%) dogs were diagnosed as being without DCM. Infected dogs with DCM exhibited structural features of LV dysfunction, e.g., a significant (*p* < 0.05) increase in the LV internal diameter at systole and diastole (LVID-s, LVID-d) and a decline in the LV posterior wall (LVPW-d) thickness at diastole. Despite an increase in stroke volume and cardiac output indicating contraction force, DCM resulted in a decreased ejection fraction, affecting systolic function. Dogs that were diagnosed as DCM-negative but were positive for *T. cruzi* by PCR exhibited a high frequency of an increase in the thickness of the interventricular septum in systole (IVS-s) and the LV posterior wall in diastole (LVPW-d), a decline in the LV inner diameter (LVID-d, LVID-s), and fractional shortening (FS). The thinning of the LVPW at systole was the most defining feature observed in chronically infected dogs. In summary, this is the first study reporting the echocardiographic changes occurring in dogs naturally infected with *T. cruzi* and developing DCM. Our data suggest that changes in LVID are a major indicator of risk of cardiac involvement, and the observation of changes in the IVS, LVPW, and FS have predictive value in determining the risk of DCM development in infected dogs.

## 1. Introduction

Chagas disease, also referred to as American Trypanosomiasis (AT), is an illness caused by a flagellated protozoan that is transmitted by hematophagous vectors. The etiological agent, *Trypanosoma cruzi*, is distributed across a large part of the American continent, and it infects several species of wild and domestic mammals and humans [1]. AT is endemic in Latin America, and an increase in the incidence of *T. cruzi* infection in dogs and humans has been noted in Mexico [2]. In Yucatan, Mexico, AT in dogs has been detected in rural and urban areas [3]. In the capital city of Yucatan, Merida, *T. cruzi* is frequently detected with a prevalence of 12.2% in apparently healthy domiciled dogs [4].

Chronic Chagas disease presents in humans as myocardial abnormalities, ranging from mild forms, such as apical aneurysm and left ventricular (LV) diastolic dysfunction only, to significant cardiac chamber dilatation, coupled with severe systolic dysfunction. Dilatation of the left and right ventricles is the most recognizable alteration of the heart in dogs and humans with Chagas disease [1,2,5,6,7,8]. Dogs are important reservoirs of *T. cruzi*, and due to the similar progression of the disease to that occurring in humans, canine AT can serve as a surrogate model for studying the course of human Chagas disease.

Electrocardiographic and echocardiographic studies in experimentally infected dogs have noted a decline in the ejection fraction is associated with thinning of the LV walls, mural thrombi, hypokinesis, and thickening of the septum [9]. More recently, echocardiography examination of seven dogs that were experimentally infected with *T. cruzi* eight years earlier identified an inversion of the E/A index, indicating a delayed relaxation pattern or mild dysfunction and hypomotility of the interventricular septum, but cardiomegaly signs were not found [10]. A detailed evaluation of the changes in cardiac structure and function in dogs naturally infected with *T. cruzi* isolates circulating in Mexico has not been carried out yet.

In this study, we aimed to characterize the echocardiographic alterations in dogs naturally infected with *T. cruzi* with ultrasound equipment normally used in routine veterinary practice. Our primary objective was to determine whether dogs naturally exposed to *T. cruzi* isolates circulating in the state of Yucatan could develop dilated cardiomyopathy and to describe the cardiac findings before the clinically severe form of the heart disease appeared in infected dogs. Our secondary objective was to obtain a comprehensive view of the changes in a range of myocardial parameters in infected dogs and determine whether these variabilities are reflective of human Chagas disease.

## 2. Materials and Methods

### 2.1. Bioethics and Study Area

All the animal studies were performed according to the protocol approved by the bioethics committee (CB-CCBA-I-2017-003) at the Facultad de Medicina Veterinaria y Zootecnia, Universidad Autónoma de Yucatán. Informed consent was obtained from the dog owners before the dogs were enrolled in the study. The study was conducted with pet dogs referred by veterinary clinics in Merida, Yucatan, Mexico (19°30″ and 21°35″ N; 87°30″ and 90°24″ W). The climate of the area is tropical sub-humid, with a well-defined rainy season during the months of May–June and October–November.

### 2.2. Selection of Animals

A cross-sectional study was carried out where 130 dogs with physical symptoms of cardiac involvement were randomly chosen. The inclusion criteria were that the dogs resided in Merida, Yucatan, with a pet owner and were seropositive for *T. cruzi* according to an ELISA. The enrolled dogs were later confirmed in terms of their *T. cruzi* exposure by Western blotting and/or PCR diagnostic approaches and had not received any treatment. The enrolled dogs (both males and females) were older than two years old and of variable sizes, weights, and breeds. Dogs were considered to have a cardiopathy when their medical history and physical exams were compatible with heart disease [11]. Electrocardiographic abnormalities, not attributable to electrolyte imbalance, were also recorded [12]. In some cases, radiographic studies or determination of blood pressure [13] were also conducted to confirm the cardiopathy. Healthy dogs (n = 16) that were seronegative for anti-*T. cruzi* antibodies and PCR-negative for *T. cruzi* DNA and exhibited no physical symptoms of cardiac involvement were used as the controls.

### 2.3. Blood Samples

From each dog, two blood samples were obtained via cephalic or jugular vein puncture. Half of each blood sample was collected in a PAXgene Blood DNA Tube (BD-QIAGEN) to preserve the DNA for later purification. A second aliquot of each blood sample was collected in a BD Vacutainer and centrifuged at 400 rpm for 15 min at room temperature to obtain the serum.

### 2.4. Serology

A serological diagnosis of AT was made according to the detection of immunoglobulins (IgGs) against *T. cruzi* by using the Chagatest ELISA recombinant v.3.0 kit (Wiener, Rosario, Argentina). The assay was carried out following the manufacturer’s recommendations, except that the 2nd antibody was replaced with horseradish peroxidase (HRP)-conjugated goat anti-dog IgGs. The details of the protocol were previously described by us [14].

### 2.5. Western Blotting

Epimastigotes of the H4 strain of *T. cruzi* were lysed in Laemmli sample buffer containing a protease inhibitor cocktail (Sigma-Aldrich, St. Louis, MO, USA). The protein samples (20 μg) were resolved on 10% polyacrylamide gels and transferred onto nitrocellulose membranes. The membranes were probed with serum samples of the dogs, and color was developed using the standard methods. A serum sample was considered positive when at least five antigenic bands were recognized [4,14].

### 2.6. PCR Detection of T. cruzi

Total DNA was extracted from whole blood samples according to a previously published protocol [4]. Alternatively, the DNeasy Blood and Tissue Kit (69504, QIAGEN, Hilden, Germany) was used to isolate the genomic DNA from the blood samples by following the manufacturer’s instructions. Total DNA was examined in terms of quality (OD_260_/OD_280_ ratio of 1.7–2.0) and quantity ([OD_260_ − OD_320_] × 50-μg/mL) by using a DU 800 UV/visible spectrophotometer. To detect the presence of *T. cruzi* DNA in the blood, a PCR assay was carried out, as described previously [3].

### 2.7. Echocardiographic Evaluation of Cardiac Structures and Function

The dogs were prepared, positioned, and scanned according to the conventional technique [15,16,17]. Mindray M5 real-time ultrasound equipment (Mindray Electronics^®^, Shenzhen, China) equipped with a cardiac transducer probe with a frequency range of 2 to 4 MHz was employed for echocardiography. The parameters of the cardiac structure and function were obtained as follows: In the right parasternal window with a short-axis view of the LV at the level of the papillary muscles, the thickness of the interventricular septum and the free walls and the diameter of the LV were measured with the M mode in systole and diastole [15,16,17]. To compare these parameters between dogs of variable sizes, weights, and breeds, the measurements of the structures were normalized according to the allometric scale formula for cardiac M-mode measurements in adult dogs [18]. Using the Teichholtz method, the software calculated the fractional shortening (FS), ejection fraction (EF), stroke volume (SV), and cardiac output (CO), which together provide an indication of LV systolic function. In the left apical position with a view of four chambers, the trans-mitral flow was measured with a pulse Doppler instrument, placing the probe at the tip of the mitral valve [19]. The peaks of the E and A waves were marked, the E/A index (a marker of LV diastolic function) was calculated, and the flow patterns were identified as normal, delayed relaxation, or restrictive, as described previously [20]. When increased blood flow velocities were observed with a normal pattern, they were surmised to be pseudo-normal, as further confirmation with a tissue Doppler was not feasible.

Dogs were considered positive for DCM according to the ultra-sonographic criteria proposed by the European Society of Veterinary Cardiology (major criteria: dilatation of the LV in systole or diastole, ventricular spheroid structure, thinning of the septum, and a reduction in fractional shortening; minor criteria: increased space between point E and the septum, incongruent values for fractional shortening and left or bilateral atrial dilatation) [21]. The reference values were based on a prediction interval of 95%, as described in the published literature [18].

### 2.8. Statistical Analysis

The normal distribution of the variables of interest was confirmed by the Shapiro–Wilk test. The evaluated animals were first grouped according to the results of the serologic and molecular tests and the presence and absence of DCM as follows: group A, serology-negative, PCR-positive, DCM-negative; Group Aw, serology-negative, PCR-positive, DCM-positive; group B, serology-positive, PCR-negative, DCM-negative; group Bw, serology-positive, PCR-negative, DCM-positive; group C, serology-positive, PCR-positive, DCM-negative; and group Cw, serology-positive, PCR-positive, DCM-positive. The control group included seronegative, PCR-negative, DCM-negative, healthy dogs (n = 16).

Echocardiographic, age, and body size data showing a normal distribution were analyzed using a one-way ANOVA, followed by Tukey’s post hoc test. The non-normally distributed data were analyzed using the Kruskal–Wallis test with Bonferroni correction, and the frequencies of the alterations in each indicator of cardiac structure and function were determined. Binary data were analyzed using the Chi^2^ test or the exact Fisher’s test to establish the association between sex, size, age, and transmitral flow patterns and a DCM-positive vs. DCM-negative disease status. All the statistical analyses were performed using Statgraphics v.19.0 software.

## 3. Results

### 3.1. Characteristics of T. cruzi Infection

All the dogs enrolled in the study were in the age range of 6.94 to 9.83 years old and of a small to medium size (weight range: 8.41–13.06 kg). PCR detection of *T. cruzi* DNA in the blood is indicative of circulating parasites and is mostly noted in response to acute infection or repeat infection. Seropositivity for anti-*T. cruzi* antibodies is generally noted in all infected cases irrespective of the stage of disease development. Thus, a lack of anti-*T. cruzi* IgGs in *T. cruzi* DNA-positive dogs indicates the very early stage of acute infection before adaptive humoral immunity has been elicited by the host. Our data showed that most of the dogs enrolled in the study were positive in both diagnostic tests, i.e., anti-*T. cruzi* antibodies by ELISA and Western blot analysis and parasite DNA detection by PCR (group C: 97 out of 130 dogs, 75% of total), indicating their exposure to repeat infections. Some dogs were PCR-positive only (group A: 26 out of 130, 20% of total), indicating the first, early phase of acute infection, and the fewest dogs were in the chronic phase (group B: 7 out of 130, 5% of total), indicated by their seropositive status only (Table 1).

Dogs were screened for anti-*T. cruzi* antibodies using two serological tests, including an enzyme-linked immunosorbent assay (ELISA) and Western blot analysis, and for circulating parasites using *T. cruzi*-specific PCR, as described in Materials and Methods. Echocardiography was performed to measure the changes in cardiac structure and function, and the animals in each group were further identified to have or not have dilated cardiomyopathy. No association was found between sex, age, or size and the presence of DCM according to the Chi^2^ or Fisher’s test.

### 3.2. Echocardiographic Findings

**Figure 1:**
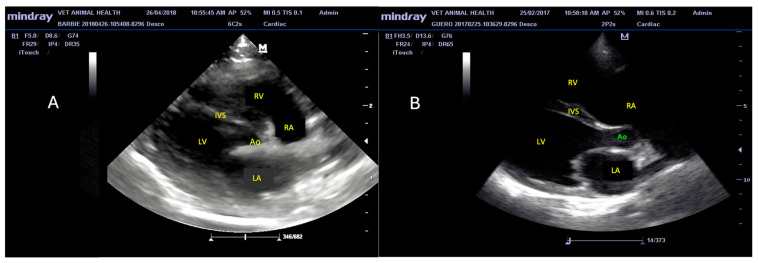
(**A**). Ultrasound (B-mode) image of the heart of a dog negative for *T. cruzi*. Chambers and septum are within the normal patterns for a healthy dog. (**B**). Ultrasound (B-mode) image of the heart of a dog with dilated cardiomyopathy (DCM) and seropositive for anti-*T. cruzi* antibodies. Shown is a right parasternal window with long-axis view of the four chambers. Dilatation of the left ventricle (spheroid shape) can be seen with thinning of the septum and its bowing towards the right ventricle. A greater diameter of the right atrium with respect to the left atrium is also noticeable. Abbreviations: RA = right atrium, LA = left atrium, RV = right ventricle, LV = left ventricle, Ao = aorta artery, IVS = interventricular septum.

Dogs were analyzed by echocardiography to measure the changes in left ventricular (LV) structure and function and characterize the presence and absence of DCM. Figure 1 shows a representative image in B mode of the cardiac characteristics of DCM in a dog positive for anti-*T. cruzi* antibodies. Note the dilatation of the LV (ventricular sphericity) with the thinning of the interventricular septum and its bowing towards the right ventricle. A greater diameter of the right atrium compared to the left atrium is also noticeable.

### 3.3. M-Mode Ultrasonographic Findings

**Figure 2:**
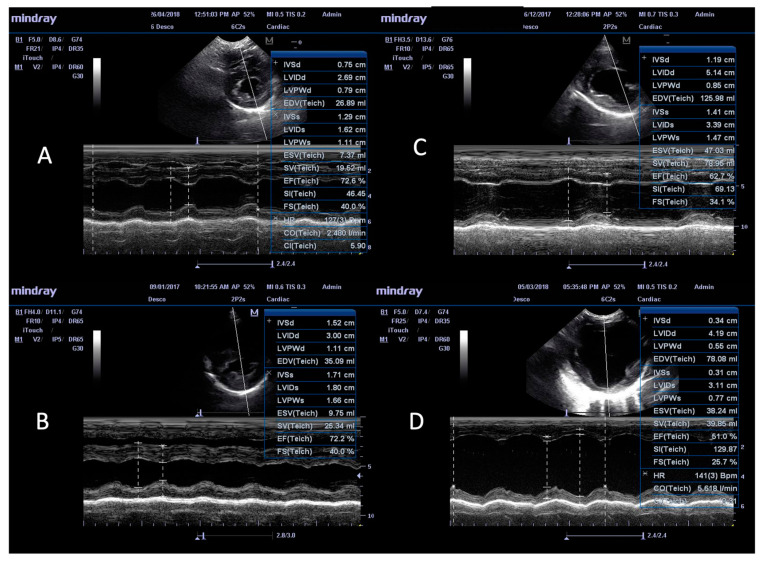
Representative images of the M-mode ultrasonographic measurement of the structures of the left ventricle in right parasternal window, short-axis view at the level of the papillary muscles, shown for infected and non-infected dogs. (**A**) Healthy, non-infected. (**B**) Seropositive for anti-*T. cruzi* antibodies and no DCM. (**C**) Seropositive with incipient DCM. (**D**) Seropositive with advanced DCM. Abbreviations: IVS = interventricular septum, LVID = left ventricle inner diameter, LVPW = left ventricle posterior wall, d = diastole, s = systole, FS = fractional shortening, EDV = end diastolic volume, ESV = end systolic volume, SV = stroke volume, EF = ejection fraction, SI= stroke index, CO = cardiac output, CI = cardiac index, HR = heart rate.

Representative M-mode ultrasonographic images and measurements of the LV structure from normal and infected dogs are shown in Figure 2. In comparison to non-infected, seronegative dogs (Figure 2A), the infected dogs with DCM (Figure 2C) and advanced DCM (Figure 2D) exhibited the highest values in terms of LV diameter, indicating the dilatation of the LV. Dogs that were infected but without DCM exhibited minor to no changes in LV diameter. Based on these structural features, we noted that 3 and 12 dogs within groups A and C, respectively, exhibited the features of DCM, while the remaining 115 infected dogs did not present with DCM (Table 1). Thus, overall, 15 out of the 130 infected dogs (i.e., 11.54%) exhibited DCM.

Next, we performed one-way ANOVA or Kruskall–Wallis analyses to determine whether the infection and seropositivity status were significantly correlated with any of the echocardiographic parameters of LV structure in dogs (Table 2A). In general, we did not observe any major changes in the IVS thickness at diastole (IVS-d) or systole (IVS-s) between any of the infected groups and the non-infected, healthy controls. The dogs exhibited an average IVS thickness of 0.4–0.5 cm at diastole and 0.64–0.70 cm at systole. The mean values of the internal diameter of the LV at diastole (LVID-d) and systole (LVID-s) were also not significantly changed in the DCM-negative dogs in groups A, B, or C (without DCM) compared to the healthy controls. However, the Aw (acute infection) and Cw (chronic and parasitemic dogs) groups with DCM exhibited a 23–40% increase in LVID-d and a 30–43% increase in LVID-s in comparison with the infected groups without DCM and the healthy controls. The mean LV posterior wall thickness at diastole (LVPW-d) was decreased by 14.9–17% in the Aw and Cw (vs. control) groups. The dogs in group B (chronic infection) exhibited a 22–28% decline in their LVPW-d and LVPW-s values (*p* < 0.05). Together, these results suggest that an increase in the LVID and thinning of the LVPW were major features of DCM in the infected dogs, and the risk of LVPW thinning was increased with chronic infection (group B).

Echocardiographic parameters of global LV function were also measured in all enrolled dogs (Table 2B). These results showed that the mean values for LV systolic function, including stroke volume (SV), heart rate (HR), and cardiac output (CO), were increased by 39–80%, 11.2–21.4%, and 62–110%, respectively, in the Aw and Cw groups compared to those noted in the infected groups (A, B, C) without DCM and the normal, healthy controls. The maximal increase in the SV, HR, and CO values was noted in the dogs in the Cw group (chronic-stage and parasitemic dogs). Despite their increased SV, the dogs in the Cw group exhibited a 15.9% decline in the ejection fraction (EF). Fractional shortening (FS) was decreased by 15% in the dogs in the Cw group. These findings indicate the early stage of DCM in the majority of the dogs in the Aw and Cw groups, as was also noted in the echocardiography imaging (Figure 2C).

### 3.4. Pulse Doppler Ultrasonographic Findings

**Figure 3:**
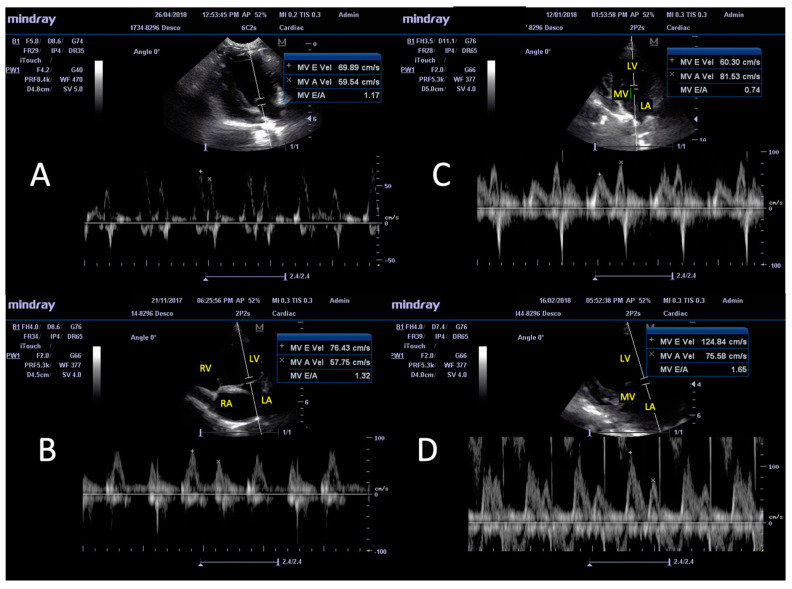
Ultrasonic recording with pulse Doppler of the transmitral blood flow and calculation of the E/A index in dogs naturally infected with *T. cruzi*. Shown is the left apical window view of four chambers. (**A**) Normal, healthy, seronegative for anti-*T. cruzi* antibodies; (**B**) pattern of delayed relaxation in a seropositive dog without DCM; (**C**) normal pattern in a seropositive dog without DCM; (**D**) flow of a restrictive pattern in a *T. cruzi*-positive dog with DCM. LA = left atrium, LV = left ventricle, RA = right atrium, RV = right ventricle, MV = mitral valve. MV E vel = velocity of peak E, MV A vel = velocity of peak A, MV E/A = index E/A.

The transmitral flow was assessed and calculation of the E/A index was carried out using ultrasonic recording with a pulse Doppler instrument. Representative images of the four chambers in the left apical window view of a seronegative healthy dog and infected dogs are presented in Figure 3, and the mean E/A values for each group are presented in Table 2. Most of the infected dogs without DCM exhibited a normal transmitral flow pattern, as was noted in the healthy controls (Figure 3A,C). A pattern of delayed relaxation (E < A) was observed in some of the infected dogs without DCM and in all the dogs with early signs of DCM (Figure 3B,D).

An increase in the velocity and time of the E and A values (Figure 3D) in *T. cruzi*-positive dogs with DCM could correspond to a pseudo-normal pattern of blood flow, but tissue Doppler imaging (TDI) is required to confirm this. At the population level, 20% of the dogs with DCM (i.e., 3 out of 15) exhibited a restrictive pattern, while a normal pattern was observed in 60% and a delayed relaxation flow was observed in 20% of the infected DCM-positive dogs. Among the infected DCM-negative dogs, 75.44% had a normal pattern, 4.39% exhibited a delayed relaxation pattern, 5.26% exhibited some features of a pseudo-normal pattern, and 14.91% exhibited a restrictive flow pattern. Though all the infected dogs exhibited an increase in the E/A ratio and the maximal increase was noted in group A (acutely infected), the average E/A values in the infected dogs were within the normal range of 0.98–1.7, and no association of the flow patterns with infection or disease status was noted using the Chi^2^ test (probability of association between type of flows and groups: 0.82). These results suggest that changes in the blood flow patterns are not directly correlated with infection and/or disease status.

We examined the frequency of alterations in the heart structure and function of the LV in infected dogs without DCM to determine whether status of infection is associated with particular features of LV dysfunction (Table 3). In group A (acute infection), 26.09% (6 out of 23) of the dogs exhibited an increase in the E/A ratio, and 21.74% of dogs exhibited a reduction in the LVID in both stages of the cardiac cycle. In group B (chronic infection), 33.3% of cases showed thinning of the LVPW at systole. In group C, the most frequent alterations were noted in LVID-s and LVID-d, which were decreased in 42.3% and 38.8% of dogs, respectively. These results suggest that changes in LVID-s and LVID-d and an increased E/A ratio are important indicators of the risk of DCM development in dogs that are acutely infected (group A) or have been repeatedly exposed to *T. cruzi* infection (group C) and are positive for *T. cruzi* by PCR. In comparison, thinning of the LV posterior wall was the most defining feature of the risk of DCM development in the chronically infected dogs (group B).

## 4. Discussion

The presence of circulating *T. cruzi* DNA in seropositive dogs (group C) may indicate a low but persistent parasitemia in chronic Chagas disease. This trend is similar to the observation made in seropositive people from endemic areas that exhibit persistence of circulating *T. cruzi* using a PCR approach [25,26]. Yucatan State, the site of this study, is a *T. cruzi*-endemic zone with abundance of the vector *Triatoma dimidiata* [27] and vector-borne transmission. Curtis-Robles et al. [28] found 25% of dogs included in their study from southern Texas were PCR-positive/seropositive for *T. cruzi*. In the present study, a larger number of cases were positive according to both tests (97 out of 130 dogs), which cannot be justified only by the presence of low, occasional parasitemia. Thus, we surmise that repeat exposure to infection contributed to the detection of circulating parasites in the seropositive dogs.

The published literature has not addressed the prevalence of DCM and heart failure in dogs. Our in-depth analysis of changes in LV structure and function in the infected dogs in this study identified a prevalence of DCM of 11.54% in the infected dogs, and the DCM features in the dogs were consistent with the clinical characterization of chronic AT made by other investigators [10,29]. The majority of the dogs included in the study were without DCM or in the early stages of DCM development. These observations do not mean that DCM is uncommon in dogs with AT but that it is a terminal feature of Chagas disease [1,2,7,30]. It is also possible that most animals do not present with this clinical condition and perhaps many may die due to arrhythmias, blockages, endocarditis, valvular endocardiosis, congestive heart failure, or other conditions that may manifest before DCM develops [8,30]. In this study, it was also found that three dogs with DCM were negative in the serological tests but positive in the PCR test, which indicates that acute *T. cruzi* infection can also result in DCM and heart failure in dogs, as is noted in 5% of acutely infected human patients. Another possibility is that these dogs were exposed to *T. cruzi* infection after they had already developed DCM from other etiologies.

Ventricular dilatation is the main feature of DCM [21], and it is recognized as one of the most well-known alterations of AT in dogs and humans [1,2,5,6,7,30]. As expected, the groups of infected dogs with DCM showed higher mean values for LVID-s and LVID-d, and these values were statistically different when compared to the other groups (Table 2). In comparison, many of the PCR-positive dogs in group A and group C exhibited a reduction in their LVID-s and LVD-d values (Table 3). This pattern is similar to that reported [31] in puppies during the acute stage of Chagas disease.

Chetboul [21] has documented the thinning of the IVS and/or the LVPW in dogs with DCM. In this study, some of the infected DCM-negative dogs exhibited increased thickening of the IVS at systole (Table 3); however, no significant changes in IVS thickness were observed in the dogs with Chagas disease (vs. healthy controls) (Table 2 and Table 3). Barr et al. [9] also documented septal thickening during systole in dogs with Chagas disease, which exceeded the maximum reference value for septal thickness. The reduction in LVPW-s seen in group B is consistent with the decrease in the size of the cardiac wall in dogs with AT reported in other studies [9].

The frequency of reduction in the LVID at systole and diastole, along with the observation of the thickening of the IVS and LVPW at systole, particularly in the PCR-positive dogs (group A and group C), suggests a tendency towards a progressive decline in the ventricular diameter. This condition has been reported in patients with acute AT [9,10,29,31,32] but not in the chronic phase of Chagas disease in experimental models and human patients. This acute left ventricular diameter reduction and septum/wall thickening of seropositive/PCR-positive dogs is possibly due to an increase in ventricular mass, as reported in mice with myocarditis [33], which in turn is produced by persistent parasitemia [26,34]. In the chronic stages, fibrosis caused by the loss of myocardial cells and their replacement by collagen fibers [35,36,37] may contribute to this reduction before DCM develops over time due to compensatory cardiovascular mechanisms [5,26]. It is also possible that there was a difference in the DTU of *T. cruzi* circulating in Merida at the time of this study with respect to previous studies [9,10,29] The *T. cruzi* DTU (TcI) circulating in this area [38] is documented to be cardiotropic and highly virulent [39,40].

Although the Teichholtz method is not optimal for determining EF, SV, and CO, it allows us to obtain values that can be obtained even using low-end ultrasound machines without specific software and by operators with little experience in echocardiography, which offers a great advantage when working in small veterinary clinics.

As noted in other studies [9,29], we recorded systolic dysfunction (decreased FS and EFs) in some of the infected animals without DCM (Table 3), though the average values for these parameters in the infected dogs (groups A, B, C) were not significantly different from those observed in the healthy controls (Table 2). This observation indicates that though they were identified as DCM-negative with normal myocardial contractility, some of the infected dogs were progressing towards the clinical development of DCM. The EF in the animals with DCM was less efficient than that in the animals without DCM despite the observation that SV and CO were significantly greater due to the dilated ventricular space. Yet there is a possibility that the increase in the systolic function parameters in the PCR-positive groups, especially in group C (Table 2), occurred due to the stress effect of the ultrasonographic procedure, which was conducted without anesthesia. Stress is documented to increase the heart rate and the ventricular contractibility transiently in small animals [41]. Further studies comparing sedated and non-sedated dogs will be needed to address the effect of stress on LV function in infected dogs.

The mean diastolic function (E/A index) was found within the reference ranges [24] in all groups (Table 2), though the E/A ratio tended to increase in all the infected groups. Blood flow patterns were not directly correlated with AT or its severity (with or without DCM) because there were similar numbers of cases with delayed relaxation and restrictive and normal patterns, with no predominance of any one parameter, in both the DCM-positive and DCM-negative infected dogs. A pattern of delayed relaxation in dogs was reported by Pascon et al. [10] in *T. cruzi*-infected dogs without DCM, but it has not been described in *T. cruzi*-positive dogs with DCM, although this and the other patterns found in the infected dogs are consistent with the pathophysiology of DCM [21].

It is particularly important to consider those indicators that were statistically different between the DCM-positive and DCM-negative groups (i.e., IVS-s, LVID-d, LVID-s, LVPW-d, and EF) because they allow for an approximation in the evaluation of these variables in dogs that are at risk of developing DCM. It should be noted that the alterations found here, when applied to an uncontrolled, randomized population, may not be associated with *T. cruzi* infection only since it is possible that there are other diseases or chronic degenerative processes that may affect the heart in a similar manner as was observed for the *T. cruzi*-infected dogs.

## 5. Conclusions

Changes in the heart structure were evident in infected dogs with DCM. Some of the infected dogs exhibited a decreased left ventricular diameter with the presence of IVS and LVPW thickening, indicating the risk of DCM development. Transmitral flow patterns were not associated with the severity of cardiac damage in the DCM-positive infected dogs. The results of the present study also suggest that changes in the LVID at systole and diastole are a major indicator of cardiac involvement in *T. cruzi*-infected dogs, and observation of changes in the thickness of the IVS and LVPW at systole or diastole, fractional shortening, and an E/A ratio above or below the normal range will provide high predictive efficacy in determining the risk of DCM progression.

## Figures and Tables

**Table 1:** Categorization of 130 dogs based on serological and molecular tests for *Trypanosoma cruzi* infection and the presence of dilated cardiomyopathy.

Dilated Cardiomyopathy	Group A Seronegative, PCR-Positive	Group B Seropositive, PCR-Negative	Group C Seropositive, PCR-Positive	Total
Absent	23	6	86	115
Present	3	-	12	15
Total	26	6	98	130

**Table 2:** Echocardiographic, age, and body size parameters in *Trypanosoma cruzi*-positive dogs.

Parameters	Mode	A (n = 23)	Aw (n = 3)	B (n = 6)	C (n = 86)	Cw (n = 12)	Controls (n = 16)
A: Structural features							
IVS diastole (IVS-d) * ^1^	M mode	0.5 ± 0.07 ^a^	0.51 ± 0.05	0.4 ± 0.08	0.49 ± 0.08 ^b^	0.4 ± 0.08 ^a,b^	0.44 ± 0.07
IVS systole (IVS-s) * ^1^	M mode	0.69 ± 0.01	0.66 ± 0.05	0.67 ± 0.17	0.7 ± 0.15	0.64 ± 0.18	0.64 ± 0.09
LVID diastole (LVID-d) * ^2^	M mode	1.44 ± 0.24 ^a^	2.02 ± 0.16 ^a,b^	1.57 ± 0.20 ^b,c^	1.35 ± 0.25 ^b,c,d^	2.05 ± 0.15 ^a,c,d,e^	1.55 ± 0.1 ^b,d,e^
LVID systole (LVID-s) * ^2^	M mode	0.83 ± 0.18 ^a^	1.15 ± 0.10 ^a,b^	0.91 ± 0.13 ^c^	0.76 ± 0.2 ^b^	1.23 ± 0.19 ^a,c^	0.86 ± 0.1 ^b^
LVPW diastole (LVPW-d) * ^2^	M mode	0.51 ± 0.09 ^a^	0.39 ± 0.16 ^a^	0.34 ± 0.03 ^a,b^	0.49 ± 0.1 ^b,c,d^	0.4 ± 0.09 ^a,c,d,e^	0.47 ± 0.06 ^b,d,e^
LVPW systole (LVPW-s) * ^2^	M mode	0.71 ± 0.09 ^a^	0.62 ± 0.18	0.53 ± 0.11 ^a,b^	0.73 ± 0.12 ^b^	0.69 ± 0.19 ^b^	0.68 ± 0.08 ^b^
EPSS ^1^ (cm)	M mode	0.39 ± 0.21^a^	0.93 ± 0.13 ^b^	0.5 ± 0.2	0.31 ± 0.21 ^b,c^	0.96 ± 0.09 ^a,c,d^	0.27 ± 0.11 ^b,d^
LA/Ao ^1^ ratio	B mode	1.39 ± 0.51 ^a^	1.69 ± 0.51	1.38 ± 0.17	1.25 ± 0.24 ^b^	1.86 ± 0.43 ^a,b,c^	1.19 ± 0.14 ^c^
SI ^1^ ratio	B and M mode	1.99 ± 0.21 ^a^	1.54 ± 0.1 ^b^	1.87 ± 0.19	1.97 ± 0.24 ^b,c^	1.53 ± 0.08 ^a,c^	1.75 ± 0.07 ^a,c^
B: Functional features							
Fractional shortening (FS), % ** ^1^	M mode	40.35 ± 6.66	40.33 ± 5.69	38.83 ± 5.03	41.39 ± 11.2	37 ± 10.72	41.75 ± 5.36
Ejection fraction (EF), % ** ^1^	M mode	72.61 ± 7.69	71.2 ± 7.30	68.33 ± 7.51	72.75 ± 12.58	64.17 ± 14.1	74.09 ± 6.68
Stroke volume (SV), mL ** ^1^	M mode	20.85 ± 14.37 ^a^	33.52 ± 14.77	27.49 ± 26.35	22.17 ± 15.5 ^b^	51.18 ± 28.4 ^a,b^	24.01 ± 10.97
Heart rate ^1^	M mode	102.43 ± 17.98	121 ± 8.54	95.33 ± 8.71	102.5 ± 31.57	121.25 ± 41.29	108.75 ± 23.95
Cardiac output (CO), L/min ** ^1^	M mode	2.1 ± 1.31 ^a^	4 ± 1.53	2.63 ± 2.52	2.22 ± 1.55 ^b^	6.6 ± 5.13 ^a,b^	2.46 ± 0.81
E/A ratio ^1^	PW Doppler	1.53 ± 0.34 ^a^	1.24 ± 0.24	1.35 ± 0.26	1.37 ± 0.34	1.41 ± 0.61	1.1 ± 0.29 ^a^
Age, years ^1^	…	9.83 ± 3.46 ^a^	7.33 ± 3.78	7.33 ± 3.33	8.52 ± 4.01	9.83 ± 2.25	5.94 ± 3.23 ^a^
Weight, kg ^1^	…	8.41 ± 6.31	9.53 ± 7.33	13.06 ± 11.86	12.77 ± 10.03	16.03 ± 18.61	9.56 ± 6.84

The Mindray© M5 ultrasound system was used to perform transthoracic echocardiography in the B and M modes and pulse wave Doppler echocardiography used to assess the changes in left ventricular diastolic function. Group A: seronegative by ELISA and Western blotting and PCR-positive, indicative of acute infection; group Aw: seronegative and PCR-positive with DCM; group B: seropositive and PCR-negative, indicative of chronic infection; group C: seropositive and PCR-positive, indicative of chronic-stage and repeat infections; group Cw: seropositive and PCR-positive for repeat infection with DCM. Controls included seronegative and PCR-negative dogs with no indication of cardiac involvement. Parameters of structural features in 1A are presented in cm. Data are presented as mean values ± standard deviation, calculated using Statgraphics software version 19. Significance (*p* < 0.05) is plotted with superscript letters, where different letters indicate statistical difference; * values were normalized as defined in Cornell et al., 2004 [18]. ** Values calculated with Teichholz formula. ^1^ Compared by Kruskal–Wallis test with Bonferroni correction, ^2^ compared by ANOVA and Tukey’s test; Abbreviations: IVS, interventricular septum; LVID, left ventricular (LV) internal diameter; LVPW, LV posterior wall; EPSS, E point septal separation; LA/Ao, left atrium/aorta; SI, sphericity index; ELISA, enzyme-linked immunosorbent assay; PCR, polymerase chain reaction; DCM, dilated cardiomyopathy.

**Table 3:** Frequency of cardiac alterations in dogs that were positive for *Trypanosoma cruzi* exposure and negative for dilated cardiomyopathy.

Parameters	Mode	A (n = 23)		B (n = 6)		C (n = 85)		Reference Range
		Increase (%)	Decrease (%)	Increase (%)	Decrease (%)	Increase (%)	Decrease (%)	
IVS, at diastole (IVS-d)	M mode	4.35	…	…	…	8.24	1.18	0.29–0.59 ^1^
IVS, at systole (IVS-s)	M mode	13.04	…	16.67	…	22.35	2.35	0.43–0.79 ^1^
LVID, at diastole (LVID-d)	M mode	…	21.74	…	16.67	…	38.82	1.27–1.85 ^1^
LVIDW, at systole (LVID-s)	M mode	…	21.74	…	…	…	42.35	0.71–1.26 ^1^
LVPW, at diastole (LVPW-d)	M mode	13.04	…	…	…	10.59	1.18	0.29–0.60 ^1^
LVPW, at systole (LVPW-s)	M mode	…	…	…	33.33	9.41	1.18	0.48–0.87 ^1^
Fractional shortening (FS)	M mode	8.70	13.04	…	16.67	22.35	20.00	33.6–49.9 ^2^
Ejection fraction (EF)	M mode	8.70	4.35	…	16.67	22.35	12.94	58.9–82.9 ^3^
E/A ratio	PW Doppler	26.09	8.70	16.67	…	11.76	3.53	0.98–1.7 ^4^

The Mindray© M5 ultrasound system was used to perform transthoracic echocardiography in the B and M modes and pulse wave Doppler echocardiography used to assess the left ventricular diastolic function. Please see Table 2 for units of measurement for each parameter: Group A: seronegative by ELISA and Western blot analysis and PCR-positive, indicative of acute infection; group B: seropositive and PCR-negative, indicative of chronic infection; group C: seropositive and PCR-positive, indicative of chronic and repeat infections. Reference cited: ^1^ Cornell et al., 2004 [18]; ^2^ Brown et al., 2003 [22]; ^3^ Serres et al., 2008 [23]; ^4^ Schober and Fuentes, 2001 [24].

## Data Availability

All the data are presented in the study. Raw data are available from the corresponding author upon reasonable request.
